# Dynamics of neural systems in epilepsy

**DOI:** 10.1186/1471-2202-14-S1-P124

**Published:** 2013-07-08

**Authors:** Kenza EL Houssaini, Viktor Jirsa

**Affiliations:** 1 UMR 1106 INSERM Institute of Systems Neuroscience, Aix-Marseille University, faculty of Medicine La Timone, Marseille, 13005, France

## 

Epilepsy is a nervous system disorder. It is characterized by a brain dysfunction activity that is expressed by an occurrence of electrical discharges at high frequency. The zone called epileptogenic region, is assumed involved in initiating seizures and whose removal is necessary for complete abolition of seizures [1]. The seizure emergence is interpreted as a bifurcation, i.e. a nonlinear transition from one state to another. A seizure transition analysis is necessary to understand the brain activity associated with this complex phenomenon, this involves making electroencephalographic (EEG) recordings.

For this aim, we study relevant experimental data of epileptic rat, the EEG shows two types of spikes:

- The first type (type 1) has bursts of high frequency spikes form.

- The second type (type 2) is characterized by a large amplitude spike followed by a slow wave.

The EEG shows that type 2 spikes amplitudes and numbers decrease when type 1 occurs. Type 1 appears just before the seizure and emerges at high frequency during the seizure (ictal phase). As time goes by, type 1 decreases regularly while type 2 increases. This experimental data analysis allows us to deduce the existence of two neuronal populations that evolve at different spatiotemporal scales.

In order to understand the mechanisms responsible for initiating seizures, we implement a mathematical approach that combines experimentation and modeling of the system involved in the generation of seizures. Regarding the modeling of seizures evolution, we rest on a model that validates these predictions. This model is a nonlinear dynamic system that responds to different emergent properties of the epileptic seizure. Due to the experimental data analysis, numerical simulations, and using mathematical tools (stability, bifurcation ...), we describe the different aspects that influence the emergence of oscillations at high frequency, therefore, the temporal evolution of the seizure.

Our mathematical model includes a set of parameters that can generate different behaviors. Specifically, we define the parameters influencing on system behavior in an important way. Then, we examine the different possible combinations of parameters in a systematic way. Subsequently, we obtain a space parameter divided into different sub-regions in which the system has a different behavior.

## Conclusion

The analysis of our mathematical model provides an explanation for invariant seizure properties seizures, and the space parameter exploration allows us to identify the important interaction between input parameters in order to determine the system behavior of each of them earlier.
